# Diagnostic value of pituitary volume in girls with precocious puberty

**DOI:** 10.1186/s12887-020-02283-7

**Published:** 2020-09-05

**Authors:** Su Wu, Yan Yang, Yujiao Wang, Qianqi Liu, Ziyang Zhu, Wei Gu

**Affiliations:** 1 Department of Endocrinology, Children’s Hospital of Nanjing Medical University, Nanjing, 210000 China; 2 Department of Radiology, Children’s Hospital of Nanjing Medical University, Nanjing, China

**Keywords:** Diagnosis, Precocious puberty, Puberty, Pituitary, MRI, Pituitary volume

## Abstract

**Background:**

To date, the gonadotropin-releasing hormone (GnRH) stimulation test is still the gold standard for precocious puberty (PP) diagnosis. However, it has many disadvantages, including low sensitivity, high cost, and invasive operation. This study aims to evaluate whether magnetic resonance imaging (MRI)-derived variables, including pituitary volume (PV), could be used as diagnostic factors for PP in girls, providing a non-invasive diagnostic approach for PP.

**Methods:**

A total of 288 young female patients who presented to the Clinic of Pediatric Endocrinology for evaluation of PP from January 2015 to December 2017 were enrolled. The sample included 90 girls diagnosed with premature thelarche (PT), 133 girls determined as idiopathic central precocious puberty (ICPP), 35 early pubertal girls, and 30 normal girls. All patients received pituitary MRI examinations.

**Results:**

The largest PV and pituitary height were shown in the ICPP and pubertal groups, followed by the PT group. The receiver operating characteristic (ROC) curve analysis showed that PV is a predictive marker for ICPP, with a sensitivity of 54.10% and a specificity of 72.20% at the cutoff value of 196.01 mm^3^. By univariate analysis, PV was positively associated with peak luteinizing hormone (LH), LH/follicle-stimulating hormone (FSH), age, bone age, and body mass index (BMI) (all *P* < 0.05). However, bone age and peak LH were the only significant predictors of PV as demonstrated by the stepwise multivariate regression analysis (Model: PV = 9.431 * bone age + 1.230 * peak LH + 92.625 [*P* = 0.000, R^2^ = 0.159]).

**Conclusions:**

The PV in the ICPP group was significantly higher than in PT and control groups, but there was no reliable cutoff value to distinguish ICPP from PT. Pituitary MRI should be combined with clinical and laboratory tests to improve the diagnostic value of PV for PP.

## Background

Precocious puberty (PP) is defined as the development of secondary sexual characteristics before the age of 9 years for boys and 8 years for girls [1, 2]. The prevalence of PP in girls is approximately ten times higher than in boys, with the estimated register-based population prevalence of below 0.05% in boys and approximately 0.2% in girls [3].

Central precocious puberty (CPP) or gonadotropin-dependent precocious puberty results from early activation of the hypothalamic-pituitary-gonadal axis (HPGA). Mutations in the kisspeptin system, including makorin RING finger protein 3 (MKRN3) and delta-like1homologue (DLK1), have been identified in sporadic and familial cases of CPP. CPP may be either idiopathic or associated with abnormalities of the central nervous system (CNS), such as hamartomas and tumors. In girls, the prevalence of idiopathic central precocious puberty (ICPP) is from 80 to 90%. In contrast, incomplete precocious puberty (IPP) develops when a secondary sexual characteristic appears, including the development of breasts and pubic hair without any hormonal changes in the HPGA. Representative types of IPP include isolated premature thelarche (PT), wherein the breasts develop, and premature adrenarche, wherein pubic and armpit hairs appear [4, 5]. CPP eventually affects the physical growth of the child with a number of adverse effects [6, 7]. Therefore, it is of great significance to distinguish CPP from common variants of PP.

The gold standard for verifying HPG activity is the response of gonadotropin to a gonadotropin-releasing hormone (GnRH) stimulation. To date, the GnRH stimulation test is still the gold standard for PP diagnosis [6, 8]. Although this test is highly specific, it has many disadvantages, such as low sensitivity, high cost, invasive operation, risk of local reaction, and unavailability of GnRH in some centers [9]. Therefore, the exploration of non-invasive diagnostic methods for CPP is urgent in the clinic.

Magnetic resonance imaging (MRI) is the currently preferred technique to image the pituitary gland and also performed in many tertiary care centers to rule out brain abnormalities for girls diagnosed with CPP [10, 11]. Since the pituitary gland enlarges at puberty, it tends to be slightly larger in height for girls (10 mm) than for boys (8 mm). Previous studies have shown that the height of the pituitary gland in the CPP group is higher than in the normal group [12, 13]. The pituitary volume (PV) has also been found to be increased with age and associated with hormonal levels [14]. However, this finding may be compromised since the incidence of these cases is rare. Besides, the relationship between PV and hormones, and the role of PV in the diagnosis of PP are still unclear.

Therefore, in this study, we aimed to evaluate the pituitary gland by MRI in PP children compared with age-matched control subjects. Meanwhile, we also investigated the impacts of MRI-derived variables on the diagnosis of PP.

## Methods

### Subjects

The research protocol of this retrospective study was approved by the Ethics Committee of the Children’s Hospital of Nanjing Medical University. The written informed consent for all the subjects to participate in this study was provided by their parents, guardians, or next of kin.

The subjects of this study were 258 young female patients who presented to the Clinic of Pediatric Endocrinology of children’s hospital of Nanjing medical university for evaluation of PP from January 2015 to December 2017. The patients associated with endocrine disorders, previous hormonal therapies, malformations, neurofibromatosis, or congenital adrenal hyperplasia were not included in this study. Finally, among enrolled 258 girls, 133 girls (mean age 6.99 years, range 2.0–8.5) were diagnosed with ICPP, 90 girls (mean age 6.88 years, range 2.5–8.5) were determined to have PT, whereas 35 girls were classified as having early puberty in clinical practice.

### Detection indicators

The patient’s age, height, weight, bone age, and laboratory testing results were collected. Body mass index (BMI) was calculated by dividing weight in kilograms by height in meters squared.

All the subjects underwent brain MRI with a detailed examination of the pituitary gland at diagnosis. The control group included 30 age-matched girls (mean age 6.90 years, range 5.0–8.0) who underwent MRI for the examination of headaches or seizures rather than breast development.

### The clinical diagnostic basis for the ICPP

The diagnosis of ICPP must conform to the diagnosis of CPP, and the pituitary MRI examination is normal. The diagnosis of CPP needs to be consistent with [15]: (1) The emergence of secondary sexual characteristics: girls 8 years old, boys 9 years old before the development. The first manifestation was the appearance of breast nodules in girls and increased testicular volume in boys. (2) Linear growth acceleration: The annual growth rate is higher than normal children. (3) Bone age ahead: advanced bone age is 1 year or more than the actual age. (4) Gonadal enlargement: Pelvic ultrasound shows that the uterus and ovaries of the girl increase in volume, and multiple follicles with a diameter of> 4 mm are seen in the ovary; the testicular volume of the boy is> 4 ml. (5) HPGA function starts, serum gonadotropin and sex hormone reach puberty level.

In this study, the diagnosis of CPP was based on the gonadotropin response to a GnRH stimulation test [16]. Patients with a peak luteinizing hormone (LH) value of >5 IU/L in the GnRH stimulation test were classified as having CPP, whereas those with a peak LH of <5 IU/L were classified as having premature thelarche (PT). Notably, the GnRH stimulation test was not performed in the control group, because the GnRH stimulation test is an invasive test and it is meaningless for the undeveloped children.

### The clinical diagnostic basis for the PT

The diagnostic criteria of PT are as follows [15]: accompanied by breast development, and is not accompanied by other signs of sexual development, no growth acceleration and early bone development, and without vaginal bleeding. The HPGA function was not activated and the basic values of serum estradiol and follicle-stimulating hormone (FSH) were often slightly increased.

### The clinical diagnostic basis for the early puberty

Diagnostic criteria for early puberty are as follows [17]: usually, the first 1–2 years of puberty and our study selects girls who have breast development after 8 years of age and the HPGA axis starts, just entering puberty.

### MRI

MRI was performed on a 1.5-T MRI (1.5 T MAGNETOM Symphony, Siemens Healthcare, Erlangen, Germany). The PV was calculated by measuring the length (L), height (H), and width (W) in millimeters of the pituitary. Length and height were determined on the midline sagittal thin section from the posterior wall to the anterior wall. The width was measured on the thin coronal section from anterior to the entrance of the pituitary stalk. Volumes were determined using the ellipsoid formula L*H*W/2 [18, 19]. The pituitary shape was visually assessed using the Elster’s grade [20], based on the contour of the gland’s superior surface in the mid-sagittal projection (grade 1 = marked concavity, grade 2 = mild concavity, grade 3 = flat, grade 4 = mild convexity, grade 5 = marked convexity). In this study, we classified the pituitary shape into 3 grades: concave (grade 1 and grade 2), flat, and convex (grade 4 and grade 5).

### GnRH stimulation testing

Regarding the GnRH stimulation test, LH and FSH levels were determined at 0, 30, 60, and 90 min after the intravenous injection of GnRH (2-3μg/kg, ≤100μg) on Roche E602 using LH electrochemiluminescence detection kit and FSH electrochemiluminescence detection kit in accordance with the kit protocols.

### Statistical analysis

Statistical analysis was performed using SPSS software, version 19.0 (SPSS Inc., Chicago). The data were shown as the mean ± standard deviation (SD). Differences among the different groups were analyzed by one-way analysis of variance (ANOVA), followed by LSD test. Statistical significance was determined as *P* < 0.05.

For evaluation of the diagnostic value of the PV, receiver operating characteristic (ROC) curve analysis was performed, in which ICPP and PT groups were the dependent variables, whereas the pituitary height, length, width, and volume were the independent variables. The optimal cutoff values were evaluated by using the Youden index (J) [21], which is defined as *J* = maximum (sensitivity + specificity − 1).

Univariate analysis was performed using the Pearson correlation coefficient for continuous variables. Stepwise multivariate regression analysis was performed using peak LH, peak FSH, LH/FSH, age, bone age, and BMI as independent variables and PV as the dependent variable.

## Results

### Comparison of clinical characteristics among PT, ICPP, and pubertal groups

As shown in Table 1, BMI was not significantly different among PT, ICPP, and pubertal groups (*P* = 0.782). Compared with the pubertal group, the peak LH, peak FSH, the ratio of peak LH to peak FSH (LH/FSH), bone age, height, and weight were significantly lower in PT group (all *P* < 0.05); the peak LH, bone age, and height were also significantly lower while the advancement of bone age over chronological age (Δage) was significantly higher in ICPP group (all *P* < 0.05).

**Table 1:** Comparison between groups for clinical characteristics

	PT	ICPP	Pubertal	*P*
Number	90	133	35	
Peak LH	3.09 ± 1.27^^^	16.27 ± 11.98***	20.22 ± 15.35#	0.000
Peak FSH	11.75 ± 4.85^	17.21 ± 8.50***	14.59 ± 6.73	0.000
LH/FSH	0.28 ± 0.14^^^	1.05 ± 0.77***	1.38 ± 0.98	0.000
Bone age (yr)	8.09 ± 1.91^^^	8.82 ± 1.74**	10.08 ± 1.30###	0.000
Δage (yr)	1.25 ± 1.26	1.83 ± 1.31**	1.20 ± 1.28#	0.001
BMI (kg/m^2^)	16.66 ± 2.03	16.49 ± 2.22	16.72 ± 1.83	0.782
Height (cm)	126.95 ± 9.35^	129.40 ± 9.70	133.56 ± 5.43#	0.001
Weight (cm)	27.12 ± 5.75^	27.86 ± 6.10	29.97 ± 5.88	0.051

Data are expressed as the mean ± SD

Data were analyzed by one-way ANOVA

Comparison between ICPP and PT, ** *P* < 0.01, *** *P* < 0.001

Comparison between ICPP and pubertal group, # *P* < 0.05, ### *P* < 0.001

Comparison between PT and pubertal group, ^ *P* < 0.05, ^^^ *P* < 0.001

PT: premature thelarche, ICPP: idiopathic central precocious puberty, LH: luteinizing hormone, FSH: follicle-stimulating hormone, Δage: bone age-chronological age, BMI: body mass index

Furthermore, the peak LH, peak FSH, LH/FSH, bone age, and Δage were significantly higher in the ICPP group than the PT group (all *P* < 0.05).

### Comparison of pituitary MRI results between control, PT, ICPP, and pubertal groups

**Fig. 1:**
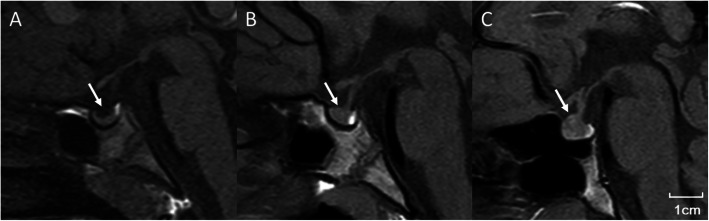
Representative magnetic resonance imaging image for concave (**a**), flat (**b**), and convex (**c**)

The MRI results showed that the pituitary length, height, and PV of the ICPP group were significantly higher than those of the PT group (all *P* < 0.05), but they were not significantly different between PT group and control group. However, the pituitary width in the PT group was significantly higher than in the control group (*P* < 0.05). Analysis of the pituitary shape demonstrated that the proportion of convexity was increased gradually in the four groups, 6.7% for the control group, 12.2% for PT, 18.8% for ICPP, and 28.6% for the pubertal group (Table 2). The representative MRI images for concave, flat, and convex were shown in Fig. 1.

**Table 2:** Comparison between groups for magnetic resonance imaging measurements

	Control	PT	ICPP	Pubertal	*P*
Number	30	90	133	35	
Age (yr)	6.90 ± 0.96	6.83 ± 1.36^^^	6.99 ± 1.33###	8.86 ± 0.37	0.000
Length (mm)	5.12 ± 1.14&&	5.43 ± 0.89	5.79 ± 0.94**	5.69 ± 0.92	0.001
Width (mm)	11.53 ± 1.35$&&	12.22 ± 1.51	12.49 ± 1.52	12.35 ± 1.44	0.016
Height (mm)	5.04 ± 0.77&	5.09 ± 1.12^	5.53 ± 1.34*	5.65 ± 1.24	0.014
PV (mm^3^)	148.72 ± 44.28&&&	170.94 ± 60.13^	200.17 ± 67.33**	200.56 ± 75.40	0.000
Grade					
concave	2	16	27	7	
flat	26	63	81	18	
convex	2	11	25	10	
Convex%	6.7%	12.2%	18.8%	28.6%	0.059

Data are expressed as the mean ± SD

Measurement data were analyzed by one-way ANOVA and count data were assessed by the chi-square test. Comparison between control group and PT, $ *P* < 0.05

Comparison between control group and ICPP, & *P* < 0.05, && *P* < 0.01, &&&*P* < 0.001

Comparison between ICPP and PT, * *P* < 0.05, ** *P* < 0.01

Comparison between ICPP and pubertal group, ### *P* < 0.001

Comparison between PT and pubertal group, ^ *P* < 0.05, ^^^*P* < 0.001

PT: premature thelarche, ICPP: idiopathic central precocious puberty, PV: pituitary volume

### Analysis of the ROC curve

**Fig. 2:**
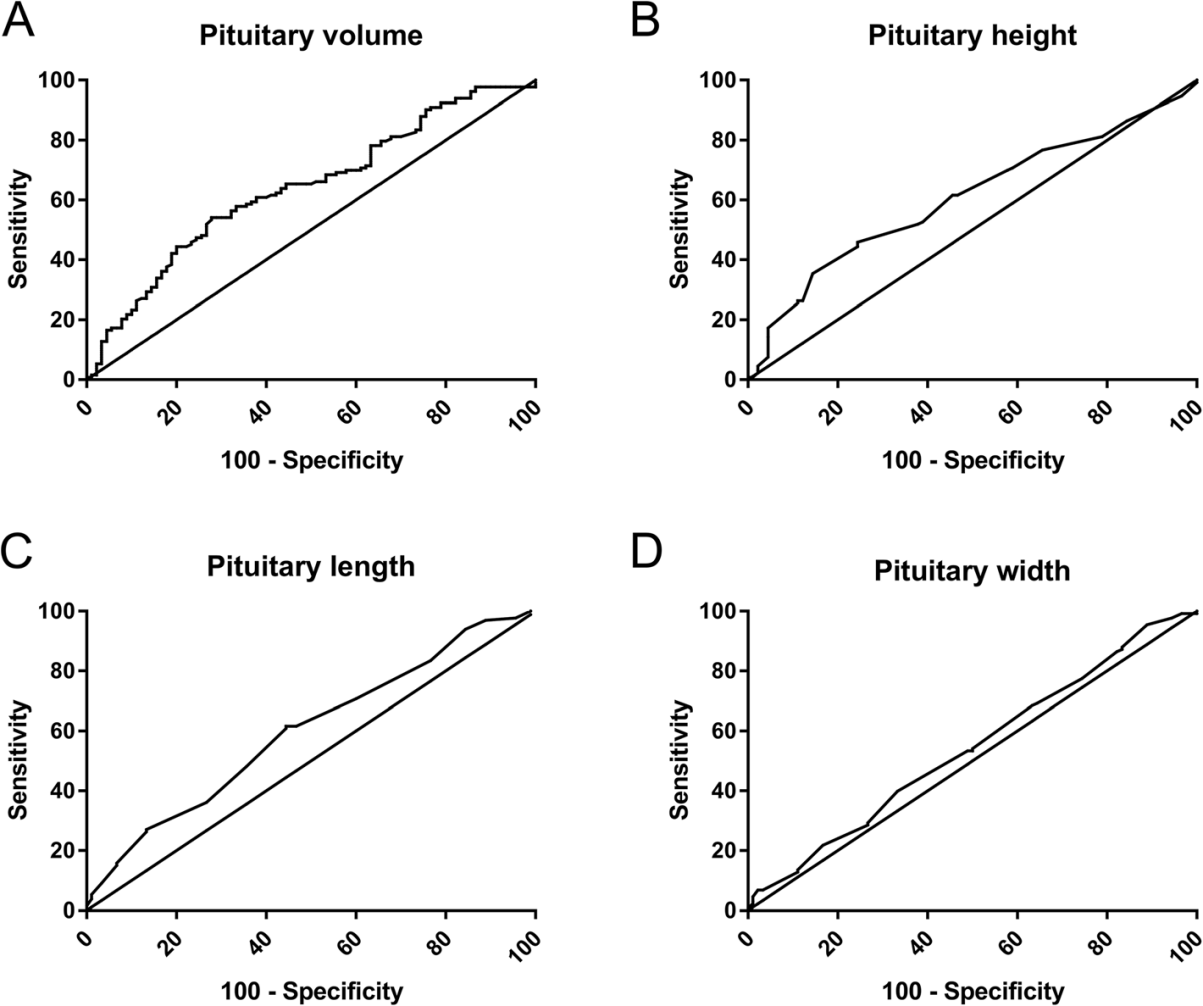
Receiver operator characteristic curves of pituitary magnetic resonance imaging measurements (pituitary volume [**a**], height [**b**], length [**c**], and width [**d**]) for the diagnosis of central precocious puberty

The area under the curve with 95% confidence interval (CI) for each MRI parameter was 0.599 (0.524–0.674) for pituitary length, 0.606 (0.532–0.680) for pituitary height, 0.541 (0.464–0.619) for pituitary width, and 0.639 (0.566–0.713) for PV. Moreover, the pituitary length showed a sensitivity of 61.70% and a specificity of 55.60% at the cutoff value of 5.445 mm. A sensitivity of 45.90% and a specificity of 75.60% at the cutoff value of 5.725 mm was observed in the pituitary height. A sensitivity of 95.50% and a specificity of 11.10% at a cutoff value of 10.25 mm was found in the pituitary width. The PV was a predictive marker for ICPP, with a sensitivity of 54.10% and a specificity of 72.20% at the cutoff value of 196.01 mm^3^ (Table 3, Fig. 2).

**Table 3:** Sensitivity, specificity, and Youden index J for several criterion values of pituitary volume

Criterion	Sensitivity(%)	Specificity(%)	Youden index J
188.37	57.90	66.70	0.246
196.01	54.10	72.20	0.263
205.70	44.40	80.00	0.244

### Correlation between PV and clinical and laboratory findings

**Fig. 3:**
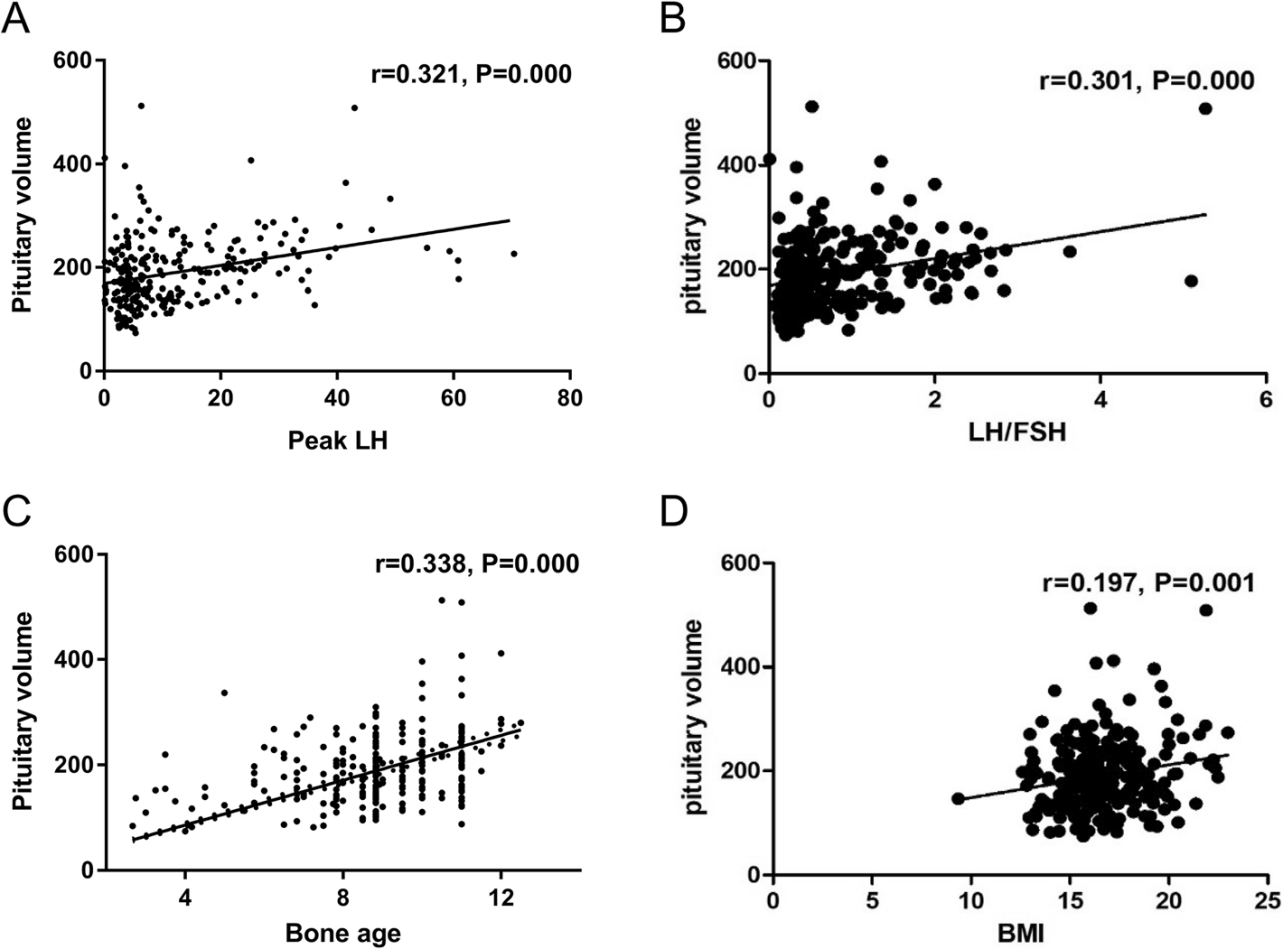
Scatterplot for the peak luteinizing hormone (LH) [**a**], LH/ follicle-stimulating hormone (FSH) [**b**], bone age [**c**], and BMI [**d**] according to pituitary volume

According to univariate analysis, we found that PV was positively associated with peak LH, LH/FSH, age, bone age, and BMI (*P* = 0.000, *P* = 0.000, *P* = 0.001, *P* = 0.000, and *P* = 0.001, respectively) (Table 4, Fig. 3). After stepwise multivariate regression analysis, the bone age and peak LH were the only significant predictors for PV with the model: PV = 9.431 * bone age + 1.230 * peak LH + 92.625 (*P* = 0.000, R^2^ = 0.159) (Table 5).

**Table 4:** Univariate analysis of the associations with pituitary volume

	r	*P*
Peak LH	0.321	0.000
Peak FSH	0.110	0.078
LH/FSH	0.302	0.000
Age	0.201	0.001
Bone age	0.338	0.000
BMI	0.197	0.001

*LH* luteinizing hormone, *FSH* follicle-stimulating hormone, *BMI* body mass index

**Table 5:** Stepwise multivariate regression analysis of the associations with pituitary volume

Model	Unstandardized Coefficients		Standard coefficient	t	P
	B	standard error			
constant	92.625	18.955		4.887	0.000
Bone age	9.431	2.239	0.260	4.212	0.000
Peak LH	1.230	0.335	0.227	3.674	0.000

All included variables are peak LH, peak follicle-stimulating hormone (FSH), LH / FSH, body mass index, bone age. LH: luteinizing hormone

## Discussion

In this study, we found that the peak LH, peak FSH, LH/FSH, bone age, and Δage were significantly higher in the ICPP group than the PT group. The pituitary length, height, and PV of the ICPP group were also significantly higher than those of the PT group, as demonstrated by the MRI examination. The PV might be a predictive marker for ICPP, with a sensitivity of 54.10% and a specificity of 72.20% at the cutoff value of 196.01 mm^3^. According to univariate analysis, PV was positively associated with peak LH, LH/FSH, age, bone age, and BMI. However, after stepwise multivariate regression analysis, the bone age and peak LH were the only significant predictors for PV. Taken together, pituitary MRI should be combined with clinical and laboratory tests to improve the diagnostic value of PV for PP.

It is difficult to distinguish ICPP and PT in the clinical diagnosis, but their differences in prognosis are extremely obvious. PT does not require treatment while ICPP has lasting adverse effects, such as short adult stature, and needs to be treated with long-acting luteinizing hormone-releasing hormone (LHRH) agonists [9, 10, 22]. Therefore, it is of great significance to distinguish the two types of PP.

Due to the disadvantages of the GnRH stimulation test, MRI has become an alternative method to evaluate the pituitary gland and to be performed in many tertiary care centers to exclude brain abnormalities in girls diagnosed with CPP. In this study, to examine the effectiveness of MRI, we compared the MRI results among the PT, ICPP, pubertal, and control groups and found significant differences in the PV and pituitary height. Patients in ICPP and pubertal groups had the largest PV and pituitary height, followed by patients with PT. In contrast, the control group had the smallest PV and pituitary height. Moreover, patients with larger pituitary glands had higher levels of LH and FSH.

CPP is accompanied by significant changes in the shape and size of the pituitary gland; patients with ICPP have a higher pituitary grade, height, and sagittal cross-sectional area compared to age-matched normal subjects [12, 13, 23]. Besides, it has been shown that the PV of CPP children is higher, and the upper pituitary surface in CPP patients appears convex in a higher proportion [23]. However, no significant differences in the pituitary length, width, and volume were observed among the control, PT, and ICPP groups. In our study, the results demonstrated that PV and pituitary height in the ICPP group were higher than those in the control group. In addition, we first reported that the PV and pituitary height of the ICPP group were higher than those of the PT group and similar to those of the pubertal group. The ratio of convexity in the pituitary gland increased in the ICPP group compared to control and PT groups without significant difference.

The stepwise multivariate regression analysis in this study built a model (R^2^ = 0.159) that explained only 15.9% of the variability in PV, even though PV showed a meaningful difference between the four groups. This phenomenon might result from the following reasons. Firstly, there was a lack of variables for generating a proper regression model, since many factors such as age, nutrition, race, sex, and the pubertal stage would influence PV [24–26]. Secondly, cells secreting gonadotrophs (LH and FSH) accounted for only 10% of the anterior pituitary cells and were distributed diffusely throughout the anterior lobe without effects on PV. Finally, the previous study showed that pituitary hypertrophy arose from the stimulation of growth-hormone-producing cells (somatotrophs) in the pituitary gland. The pituitary enlargement in puberty might also be correlated with the serum levels of somatomedin C [12].

Besides, our data indicate that a larger PV is correlated with a higher peak LH value, but not with the peak FSH value. It has been shown that a larger PV is associated with a higher FSH production but is independent of pubertal development in normal subjects [14]. However, in this study, the positive association between LH and PV is linked to pubertal development. The previous study has demonstrated that the gradually elevated FSH had been already ongoing for several years prior to the onset of puberty [27]. Therefore, we propose that increased LH levels are associated with a larger pituitary gland during early puberty, whereas the association between peak FSH and PV is not obvious in the early adolescence.

We also conducted the univariate logistic regression analysis with each MRI parameter as an independent variable to evaluate the diagnostic value of pituitary MRI in CPP girls. The PV was a predictive marker for ICPP, with a sensitivity of 54.10% and a specificity of 72.20% at the cutoff value of 196.01 mm^3^. However, these results did show a reliable predictor. In general, the cutoff values obtained by ROC curves showed low sensitivity and specificity, even for the most potential predictor, PV.

This study had several limitations. Firstly, this was a retrospective study which might cause the loss of some clinical data. Secondly, we did not collect basal LH, basal FSH, E2, and Tanner stage. Thirdly, only one experienced radiologist performed all imaging studies. In addition, although the sample size of this study is larger than that of the previous studies [12, 13], the sample size is still relatively small considering the incidence of precocious puberty. The clinical manifestations of polycystic ovary syndrome (PCOS) patients mostly start in puberty and the main clinical features are particularly similar to physiological changes in puberty, such as insulin resistance [28, 29]. CPP has been shown to be the first appearance of PCOS [30]. Our study did not include other comprehensive analyses such as ovarian color, Doppler ultrasound and other results, which are required to be assessed in the future.

## Conclusions

The PV in the ICPP group was significantly higher than that in PT and control groups, but there was no reliable cutoff value to distinguish ICPP from PT. Pituitary MRI should be combined with clinical and laboratory tests to improve the diagnostic value of PV for PP, providing a non-invasive diagnostic method for PP.

## Data Availability

The analyzed data sets generated during the study are available from the corresponding author on reasonable request.

## Abbreviations

PP: Precocious puberty
CPP: Central precocious puberty
HPGA: Hypothalamic-pituitary-gonadal axis
MKRN3: Makorin RING finger protein 3
DLK1: Delta-like1homologue
CNS: Central nervous system
ICPP: Idiopathic central precocious puberty
IPP: Incomplete precocious puberty
PT: Premature thelarche
GnRH: Gonadotropin-releasing hormone
MRI: Magnetic resonance imaging
PV: Pituitary volume
BMI: Body Mass Index
LH: Luteinizing hormone
ET: Exaggerated thelarche
FSH: Follicle-stimulating hormone
L: Length
H: Height
W: Width
SD: Standard deviation
ANOVA: Analysis of variance
ROC: Receiver operating characteristic
J: Youden index
CI: Confidence interval
LHRH: Luteinizing hormone releasing hormone
PCOS: Polycystic ovary syndrome
